# Correlation analysis on serum inflammatory cytokine level and neurogenic pulmonary edema for children with severe hand–foot–mouth disease

**DOI:** 10.1186/s40001-018-0313-1

**Published:** 2018-05-03

**Authors:** Jin-Fang Sun, Hao-Lan Li, Bao-Xia Sun

**Affiliations:** Department of Infections Disease, Zaozhuang Municipal Hospital, Leading Road No. 41, Shizhong District, Zaozhuang, 277100 Shandong China

**Keywords:** Hand–foot–mouth disease, Cytokines, Dynamic change, Neurogenic pulmonary edema

## Abstract

**Objective:**

This study aims to discuss the correlation between serum inflammatory cytokines and neurogenic pulmonary edema (NPE) in children with severe hand–foot–mouth disease (HFMD).

**Methods:**

A total of 89 patients with severe HFMD were enrolled into this study. These patients were divided into two groups, according to the presence of NPE: central nervous system disease (CNSD) group and NPE group. Serum IL-4, IL-10, IL-6, IL-17, tumor necrosis factor-α (TNF-α), and interferon-γ (IFN-γ) levels were measured in patients by enzyme-linked immunosorbent assay at 1, 3, and 5 days after admission. Furthermore, risk factors for NPE were screened using multivariable logistic regression analysis.

**Results:**

IL-6, TNF-α, IL-10, and interferon-γ (IFN-γ) levels in the NPE group were higher than in the CNSD group. TNF-α, IL-10, and IFN-γ levels reached a peak on the 3rd day of admission. Age, continuous fever, blood sugar, white blood cell count, and IL-10 were risk factors for the occurrence of NPE in severe HFMD.

**Conclusion:**

The dynamic unbalance of inflammatory cytokines is related to the occurrence and progress of NPE.

## Background

Neurogenic pulmonary edema (NPE) is an important complication and a primary cause of death in hand–foot–mouth disease (HFMD), and most of these cases die within 12 h after onset [1]. Research has shown that serum inflammatory cytokine levels significantly increase in severe HFMD patients with NPE [2–4], and is closely correlated to its prognosis [5, 6]. However, there is lack of research on the dynamic change in serum inflammatory cytokines and its correlation to NPE in patients with severe HFMD during the progress of the disease. In the present study, the dynamic changes in inflammatory cytokine levels in patients with severe HFMD after admission, who were treated and cured in two hospitals in Henan Province, China from March 2010 to December 2012, were monitored. Furthermore, the correlation between serum inflammatory cytokines and NPE in patients with severe HFMD, and the indicators for predicting NPE, were discussed to provide basis for disclosing the formation mechanism of these severe cases.

## Methods

### General information

Case selection: pediatric patients with clinically diagnosed severe HFMD, who were admitted at Zhengzhou Children’s Hospital and Henan Infectious Hospital from March 2010 to December 2012, were included into the present study. Severe HFMD was clinically diagnosed according to the clinical characteristics, including fever with or without rash, and neurological, respiratory, and circulatory manifestations. All patients were identified and treated according to the *HFMD Medical Technology Guidance for Medical Institutions* (issued by the Ministry of Public Health in 2008) [7]. A signed informed consent was obtained from the parents of children who were clinically diagnosed with severe HFMD and hospitalized, in order to collect specimens from admission. This study was approved by the Medical Ethics Committee of these two hospitals. The treatment for these patients was a combination of intravenous immune globulin (IVIG) and corticoids: IVIG, *qd*, 1.0 g/kg at a time, continuous use for 2 days; corticoids, *bid*, 2.0 mg/kg at a time, the dose was halved after 3 days, and the treatment was stopped after 2 days.

### Patients grouping

According to the occurrence of NPE, the severe HFMD children were divided into two groups: NPE group and central nervous system disease (CNSD) group. The diagnosis criteria for NPE: (1) emergency dyspnea, cyanosis, respiration rate > 30 breaths per minute, frothy sputum; (2) both lungs have visibly moist rales or howling; (3) chest X-ray revealed increased bronchovascular shadows, a typical butterfly shape, or plaque in the lung; (4) arterial blood gas analysis revealed low PaO_2_ and high PaCO_2_; (5) no primary heart, lung, or kidney disease; (6) cardiac pulmonary edema and left heart failure was excluded, which was caused by cardiogenic pulmonary edema, and too fast and excessive infusion. CNSD was diagnosed on the basis of the absence of pulmonary edema. Major criteria for neurological involvement: (1) poor mental health, lethargy, headache and vomiting, and body shaking, muscle clonus, nystagmus, ataxia, delayed paralysis, convulsion, etc.; (2) the cerebrospinal fluid examination revealed a clear appearance, increased pressure, increased white blood cell count, mostly mononuclear cells, normal or slightly increased protein level, and normal sugar and chloride levels; (3) MRI revealed that the brainstem and spinal cord gray matter were damaged. The difference in the duration of the disease between the two groups was 1 day.

### Data acquisition

Respiration, pulse, blood pressure, and temperature measurements were first collected within 6 h of patient admission. Laboratory inspection results were recorded within 72 h after admission for the first detection. Relevant scales, and clinical classifications and evaluations were respectively conducted by two experienced pediatricians. Furthermore, the disease changes in patients were recorded daily.

### Inflammatory cytokine detection

On the 1st, 3rd, and 5th day after admission of the patient, the serum IL-6, TNF-α, IL-4, IL-10, IL-17, and IFN-γ levels of each child were determined using an enzyme-linked immunosorbent assay detection kit (Shanghai Joyee Biotechnics Co., Ltd.), according to kit introductions.

### Statistical analysis

For the comparison of continuous variables, a normality test was performed using the Kolmogorov–Smirnov test. Variables that conform to normal distributions were expressed as $\bar{x} \pm s$, otherwise were expressed as medians (such as p25 and p75). Data were verified using calibrated-t, and measurement data that did not conform to normal distributions were analyzed using the Mann–Whitney *U* test. Count data were presented as a frequency number (%) and evaluated using *χ*^2^ test. For repeated measurement data, analysis of variance (ANOVA) was used for the comparisons made between or intra-groups. Multivariable analysis was performed using the Logistic regression forward-stepwise method, the variables were disregarded according to the result of the likelihood-ratio test, and risk factors were screened with an significance level of 0.05 for inclusion and significance level of 0.1 for exclusion. All data were analyzed using SPSS 19.0, and the inspection level was set at *α* = 0.05 on both sides.

## Results

### General information

In the 89 severe HFMD children, NPE occurred in 42 children (NPE group) and CNSD occurred in 47 children (CNSD group). For the comparison of age, length of hospital stay, time of fever duration, heart rate, positive rate of enterovirus type 71 (EV71), white blood cell count, and blood sugar, there was a significant difference in aspects such as blood sugar (*P *< 0.05, Table 1).

**Table 1:** Comparison on demographic and clinical data for NPE and CNSD children patients

	NPE group	CNSD group	*P* value
*N*	42	47	0.015
Age (months, $\bar{x} \pm s$)	17.55 ± 5.12	22.94 ± 10.37	0.552
Boy (cases, (%))	23 (54.76)	30 (63.83)	0.257
Body mass (kg, $\bar{x} \pm s$)	11.86 ± 2.02	12.72 ± 2.71	
Residence			0.267
City	10 (23.81)	13 (27.66)	
Rural area	22 (52.38)	22 (46.81)	
Rural–urban fringe zone	10 (23.81)	12 (25.53)	
Length of stay (days) median (P25 and P75)	8 (6,9)	6 (5,7)	0.000
Maximum body temperature (°C, $\bar{x} \pm s$)	38.89 ± 0.60	38.73 ± 0.80	0.480
Duration of fever (days) median (P25 and P75)	4 (4,5)	3 (2,4)	0.000
Cardiac rhythm (times/min, $\bar{x} \pm s$)	227.21 ± 28.23	161.09 ± 23.31	0.000
EV71-positive cases (%)	37 (88.10)	29 (61.70)	0.024
White blood cell count (×109/L, $\bar{x} \pm s$)	13.75 ± 3.68	12.84 ± 2.78	0.033
CRP (mg/L, $\bar{x} \pm s$)	15.43 ± 10.31	9.83 ± 6.28	0.898
Blood sugar (mmol/L, $\bar{x} \pm s$)	7.58 ± 1.83	6.16 ± 1.14	0.003
IVI therapy cases (%)	37 (88.10)	38 (80.85)	0.786
Hormonal therapy cases (%)	40 (95.24)	43 (91.49)	0.901

CNSD, central nervous system disease; NEP, neurogenic pulmonary edema; CPR, C-reactive protein; EV71, human enterovirus 71; IVIG, intravenous immune globulin

^a^*t* test; ^b^ χ^2^ test; ^c^ Mann–Whitney *U* test

### The dynamic changes in inflammatory cytokines

Serum IL-6, TNF-α, IL-10, and IFN-γ levels were higher in the NPE group than in the CNSD group, and the differences were statistically significant (*P *< 0.05). In addition, serum TNF-α, IL-10, and IFN-γ levels in the NPE group reached the peak value at the 3rd day of admission (Table 2).

**Table 2:** Dynamic change for serum inflammatory cytokines of NPE and CNSD children patients

Group	Cases	IL-6	TNF-α	IL-4	IL-10	IL-17	IFN-γ
Group NPE							
1 day after admission	42	70.56 ± 17.20	58.19 ± 20.67	20.24 ± 5.13	64.11 ± 19.14	55.67 ± 42.18	56.61 ± 10.94
3 days after admission	42	74.80 ± 20.68	52.74 ± 20.22	22.83 ± 3.63	80.14 ± 19.20	53.33 ± 25.22	58.42 ± 9.17
5 days after admission	42	63.73 ± 23.85	30.08 ± 12.20	21.28 ± 4.50	59.92 ± 20.95	48.10 ± 27.22	55.31 ± 8.94
Group CNSD							
1 day after admission	47	65.70 ± 18.27	54.57 ± 22.01	19.05 ± 5.78	59.56 ± 22.85	48.14 ± 33.26	48.91 ± 11.73
3 days after admission	47	55.99 ± 13.86	33.57 ± 11.43	20.38 ± 4.54	59.68 ± 16.98	44.81 ± 28.44	53.11 ± 8.96
5 days after admission	47	49.23 ± 13.34	28.92 ± 10.09	19.60 ± 4.54	42.12 ± 16.52	34.29 ± 27.33	45.88 ± 11.06
Value *i* *F*_interblock_ value (*P* value)		7.855 (0.077)	3.644 (0.042)	4.361 (0.052)	16.823 (0.000)	3.061 (0.084)	20.142 (0.000)
*F*_time_ value (*P* value)		1.876 (0.177)	13.133 (0.001)	0.873 (0.354)	14.580 (0.001)	1.329 (0.254)	3.093 (0.045)
*F*_interaction_ value (*P* value)		2.192 (0.145)	0.2919 (0.592)	0.152 (0.618)	2.612 (0.078)	3.736 (0.059)	0.513 (0.600)

CNSD, central nervous system disease; NPE, neurogenic pulmonary edema; *F*_interblock_ value, It refers to the variation (*F* value) between NPE group and CNSD group; *F*_time_ value, it refers to the variation of different time (1, 3, 5 days) about NPE group or CNSD group; *F*_interaction_ value, it refers to the interaction variation of group and time. ANOVA of repeated measurement data was used for the statistical test about the comparisons made between or intra-groups

TNF-α level was higher in the NPE group (52.74 ± 20.22) than in the CNSD group (33.57 ± 11.43) at the 3rd day of admission, and the difference between these two groups at the 1st (NPE group: 58.19 ± 20.67; CNSD group: 54.57 ± 22.01) and 5th (NPE group: 30.08 ± 12.20; CNSD group: 28.92 ± 10.09) day was not statistically significant. Furthermore, the time to peak duration was shorter in the NPE group. In addition, IL-6 (d1: NPE group: 70.56 ± 17.20, CNSD group: 65.70 ± 18.27; d3: NPE group: 74.80 ± 20.68, CNSD group: 55.99 ± 13.86; d5: NPE group: 63.73 ± 23.85, CNSD group: 49.23 ± 13.34) and IFN-γ (d1: NPE group: 56.61 ± 10.94, CNSD group: 48.91 ± 11.73; d3: NPE group: 58.42 ± 9.17, CNSD group: 53.11 ± 8.96; d5: NPE group: 55.31 ± 8.94, CNSD group: 45.88 ± 11.06) were higher in the NPE group than in the CNSD group within 5 days from admission. The IL-10 level reached a peak value at the 3rd day of admission, and the difference between these two groups at the 1st day was not statistically significant. However, this was observed to increase at the 3rd and 5th day. IL-10 mainly has an anti-inflammatory effect in inhibiting the synthesis of inflammatory mediators such as TNF-α and IL-6 after activation.

### Multivariable logistic analysis

Risk factor evaluation using an unconditional logistic regression equation in a one-time manner and the interactions among the factors were used to screen risk factors for NPE occurrence in several severe patients, and the integrated effects of multiple factors on the absolute risk of NPE were estimated (forward-stepwise regression method). Finally, age, continuous fever, white blood cell count, blood sugar, and IL-10 levels were included into the outcome model (Table 3). The simulated fitting degree analysis (Hosmer–Lemeshow detection) revealed that the fitting degree of the model was good (*χ*^2^ = 5.943, *P *= 0.546).

**Table 3:** Multivariate logistic regression analysis on the risk factors of severe hand–foot–mouth disease children with neurogenic pulmonary edema

Variable	SE	OR value	95% CI	*P* value
Constant term	0.751			0.000
Age (≤ 24 months)	0.541	3.383	1.173–4.799	0.024
Duration of fever (> 3 days)	0.526	4.925	1.758–3.794	0.002
White blood cell count (> 15.0 × 10^9^/L)	0.499	3.465	1.303–5.220	0.013
Blood sugar (> 8.3 mmol/L)	0.560	7.579	2.530–12.704	0.000
IL-10 (> 60.00 eg/L)	0.110	1.228	1.007–1.523	0.037

IL, interleukin; OR, odds ratio; 95% CI, 95% confidence interval

## Discussion

NPE is the most severe complication for severe HFMD. Immune research has indicated that HFMD patients, who have a slight symptom, are in a systemic inflammatory response state, and that severe or critical patients are in a compensatory anti-inflammatory response or mixed antagonistic response state [8–10]. Furthermore, inflammatory cytokine levels in HFMD patients with NPE significantly increase, and are closely correlated with the prognosis [2–6]. TNF-α and IL-6 are proinflammatory mediators [11–13]. TNF-α can cause the “waterfall” inflammatory response of inflammatory mediators, and IL-6 plays a collaboration and amplification role in inflammatory response. The early production of IL-10 is relatively insufficient, which causes TNF-α and IL-6 to be difficult to control at continuously high levels. Furthermore, it also induces inflammatory responses in tissues and organs. However, in the later period, IL-10 continuously increases to enter into the compensatory anti-inflammatory response, which inhibits the immunologic functions of the body. Furthermore, the capability of removing pathogenic microorganisms is decreased. Therefore, IL-10 may have a double-sided effect in severe HFMD disease progress and changes. Furthermore, this is one of the important reasons that cause the progress of the HMFD state, which conforms to the results of domestic and overseas studies [14, 15].

The disease of an NPE child rapidly progresses, and the fatality rate increases when respiratory circulatory failure occurs. Furthermore, even though they survive, the sequela increases. At present, there is a lack of safe and effective vaccines for humans, globally, and there is no antiviral drug with a definite effect. Therefore, it is the key to increase the cure rate and improve the prognosis in severe patients by identifying the main risk factors, and taking corresponding cure measures timely during the early period. In a number of studies, early severe patients have been identified by comparing clinical and laboratory examinations for general and severe HFMD patients [16–18]. In the present study, the risk factors for NPE included the following: age of ≤ 24 months old, continuous fever, elevated heart rate, Ev71 positivity, elevated peripheral blood leucocyte and blood sugar levels, and the continuous increase in inflammatory cytokines (IL-6, TNF-α, IL-10, and IFN-γ). These conform to the results of previous studies [16–18]. The occurrence of NPE in HFMD is closely correlated to brainstem injury [19]. After brainstem injury, it may cause the dysfunction of autonomic nerves and stress response in the body. Autonomic nerve dysfunction may cause vomiting, convulsion, changes in respiratory rhythm, elevated blood sugar, and hyperleukocytosis. When the body is in a stressed state, it may cause the release of multiple active substances from the internal and external brain, sympathetic nerve excitation, release of much catecholamine, and the waterfall-like release of inflammatory mediators. Furthermore, it can also cause permeability to increase in the pulmonary vessel endothelium and NPE [20]. In the present study, multivariable logistic recession analysis was conducted on variables with statistical significance in a single-factor analysis. The analysis revealed that age, continuous fever, peripheral blood leucocyte, blood sugar, and IL-10 are independent risk factors for the occurrence of severe HFMD.
