# Correction to: Correlation analysis on serum inflammatory cytokine level and neurogenic pulmonary edema for children with severe hand–foot–mouth disease

**DOI:** 10.1186/s40001-018-0327-8

**Published:** 2018-06-15

**Authors:** Jin-Fang Sun, Hao-Lan Li, Bao-Xia Sun

**Affiliations:** Department of Infections Disease, Zaozhuang Municipal Hospital, Leading Road No. 41, Shizhong District, Zaozhuang, 277100 Shandong China

## Correction to: Eur J Med Res (2018) 23:21. 10.1186/s40001-018-0313-1

The original publication of this article [1] contained two erroneous paragraphs related to the time and place for the admission of the pediatric patients with clinically diagnosed severe HFMD. The updated information has been indicated in **bold.**

- **Incorrect:** In the present study, the dynamic changes in inflammatory cytokine levels in patients with severe HFMD after admission, who were treated and cured in two hospitals in Henan Province, China from March 2010 to December 2012, were monitored.
- **Correct:** In the present study, the dynamic changes in inflammatory cytokine levels in patients with severe HFMD after admission, who were treated and cured in two hospitals in **Shandong** Province, China from March **2012** to December **2016**, were monitored.
- **Incorrect:** Case selection: pediatric patients with clinically diagnosed severe HFMD, who were admitted at Zhengzhou Children’s Hospital and Henan Infectious Hospital from March 2010 to December 2012, were included into the present study.
- **Correct:** Case selection: pediatric patients with clinically diagnosed severe HFMD, who were admitted at our hospital from March **2012** to December **2016**, were included into the present study.
